# Vulnerable individuals and changes in suicidal behaviour during the COVID-19 pandemic in Korea

**DOI:** 10.1192/bjo.2022.569

**Published:** 2022-09-08

**Authors:** Gi Eun Lee, Jong Won Kim, Kyeong Ryong Lee, Dae Young Hong, Sang O Park, Sin Young Kim, Kwang Je Baek, Hong Jun Jeon

**Affiliations:** Department of Emergency Medicine, School of Medicine, Konkuk University, Konkuk University Medical Center, Republic of Korea; Department of Psychiatry, School of Medicine, Konkuk University, Konkuk University Medical Center, Republic of Korea

**Keywords:** Suicide attempt, mental health, COVID-19, risk factors, Korea

## Abstract

**Background:**

The COVID-19 pandemic poses a major threat to mental health and is associated with an increased risk of suicide. An understanding of suicidal behaviours during the pandemic is necessary for establishing policies to prevent suicides in such social conditions.

**Aims:**

We aimed to investigate vulnerable individuals and the characteristics of changes in suicidal behaviour during the COVID-19 pandemic.

**Method:**

We retrospectively reviewed the medical records of patients with suicide attempts who visited the emergency department from February 2019 to January 2021. We analysed the demographic and clinical characteristics, risk factors and rescue factors of patients, and compared the findings between the pre-pandemic and pandemic periods.

**Results:**

In total, 519 patients were included. During the pre-pandemic and pandemic periods, 303 and 270 patients visited the emergency department after a suicide attempt, respectively. The proportion of suicide attempts by women (60.1% *v*. 69.3%, *P* = 0.035) and patients with a previous psychiatric illness (63.4% *v*. 72.9%, *P* = 0.006) increased during the COVID-19 pandemic. In addition, patients’ rescue scores during the pandemic were lower than those during the pre-pandemic period (12 (interquartile range: 11–13) *v*. 13 (interquartile range: 12–14), *P* < 0.001).

**Conclusions:**

Women and people with previous psychiatric illnesses were more vulnerable to suicide attempts during the COVID-19 pandemic. Suicide prevention policies, such as continuous monitoring and staying in touch with vulnerable individuals, are necessary to cope with suicide risk.

Every year, more than 700 000 people worldwide die by suicide.^1^ Suicide is the most common cause of death among adolescents and young adults aged 10–29 years.^2^ The aggregate lifetime prevalence of suicide attempts, suicide plans and suicidal ideation are 6%, 9.9% and 18%, respectively.^3^ Korea has a very high rate of suicides and ranks first among the Organisation for Economic Co-operation and Development member countries in this regard.^4^ Suicide attempts can place physical, psychological and economic burdens on the patients attempting suicide, as well as their families and communities. Therefore, suicide is a critical social and public health concern.

## A variety of factors

Suicide attempts are associated with a variety of factors, including biological, psychological, social and environmental factors.^5^ Psychiatric illnesses, such as depression or alcohol use disorder, physical illnesses, ethnic differences, gender and age, are all well-known risk factors for suicide attempts.^6–10^ Conflicts in interpersonal relationships, feelings of isolation or economic problems are also linked to suicide attempts.^11–13^ The COVID-19 pandemic forced the implementation of lockdown restrictions, including social distancing and shelter-in-place orders, to prevent the spread of infection. However, several people experienced anxiety and depression owing to the stress associated with COVID-19 countermeasures. Several studies have shown that social distancing and strong quarantine resulted in a decline in quality of life along with the disruption of daily life, which eventually exacerbated psychological stress.^14,15^ They also faced economic stress caused by economic downturns.^16^ These potential threats may have a significant influence on suicidal behaviours. Recent studies have demonstrated that the COVID-19 pandemic is associated with suicide mortality as well as suicidal ideation and attempts.^17,18^ However, other studies conducted in the early phase of the COVID-19 pandemic indicated either a reduction or no change in suicidal behaviour. Therefore, additional research is needed to resolve this inconsistency.

Suicide can be prevented through timely intervention by diverse members of society, such as government, community and healthcare professionals. In Canada, an overall decrease in suicide mortality has been reported because of timely government intervention, such as reducing measures of insecurity and providing psychiatric services during the COVID-19 pandemic.^19^ Suicide prevention requires recognition of warning signs and reducing risk factors in vulnerable people. An understanding of the influence of changes in the external environment on suicide attempts can facilitate planning of suicide prevention activities. The purpose of this study was to investigate the influence of the COVID-19 pandemic on suicidal behaviour. We compared the clinical characteristics of suicide attempts, including the risk–rescue rating in the emergency department during the COVID-19 pandemic period, in comparison with those during the pre-pandemic period.

## Method

### Study design and participants

In this retrospective study, we reviewed the medical records of all people who had attempted suicide and visited the emergency department of a tertiary hospital in Seoul, Korea, from February 2019 to January 2021. The study period was divided into a pre-pandemic period (February 2019 to January 2020) and a pandemic period (February 2020 to January 2021). All patients were primarily evaluated by emergency physicians and then interviewed by a psychiatrist; if the patients could not be interviewed, their guardian was interviewed instead. Missing data were excluded. The authors assert that all procedures contributing to this work comply with the ethical standards of the relevant national and institutional committees on human experimentation and with the Helsinki Declaration of 1975, as revised in 2008. All procedures involving human patients were approved by the Institutional Review Board of Konkuk University Medical Center (reference KUMC2022-04-029). Informed consent was waived because of the retrospective nature of the study.

### Measures

Patients’ baseline demographic factors (age, gender, employment status, religious status, household members and education level) and clinical factors (physical illness, previous psychiatric history, previous suicide attempt history and reason for suicide attempt) were collected. The reason for a suicide attempt was the primary reason for suicidal behaviour in patients as identified by a psychiatrist's interview in the emergency department. It was categorised into conflict with others, psychiatric problems, economic problems, physical illness and other (impulsive, alcohol-related and unknown). The status of the patients after emergency department treatment was categorised into discharge, admission or transfer to another hospital. The risk–rescue rating in the suicide assessment^20^ was calculated with the Risk–Rescue Rating Scale to evaluate lethality; it is well-known as a reliable, quantitative measure of lethality. The risk factors consisted of agent used, impaired consciousness, lesions/toxicity, reversibility and treatment required. The rescue factors consisted of location, person initiating rescue, probability of discovery by any rescuer, accessibility of rescue and delay until discovery. Each of five risk factors and five rescue factors was rated on a three-point scale, and then the total score was converted into overall risk and rescue scores ranging from 1 to 5. Mortality and intensive care unit admission data were also collected.

### Statistical analysis

We conducted the Mann–Whitney *U*-test to compare continuous variables and the chi-squared test to compare categorical variables between patients who visited the emergency department for suicide attempts during the pre-pandemic and pandemic periods. Continuous variables are presented as medians with interquartile ranges (IQRs). and categorical variables are presented as numbers with percentages. Statistical significance was set at *P* < 0.05. All statistical analyses were performed with SPSS for Windows version 19 (SPSS Inc, Chicago, USA).

## Results

**Fig. 1:**
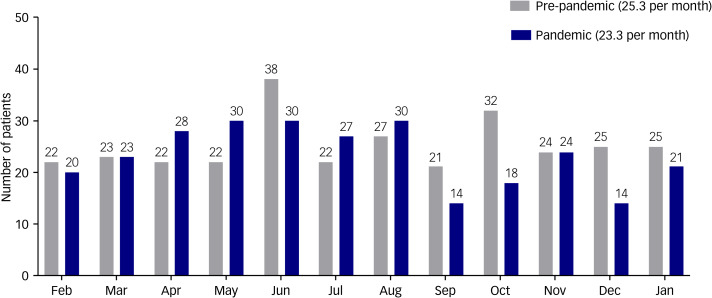
The number of patients attempting suicide per month during the pre-pandemic (February 2019 to January 2020) and pandemic (February 2020 to January 2021) periods. In the pre-pandemic period, an average of 25.3 patients per month (303 total) presented to the emergency department after a suicide attempt, whereas 23.3 patients per month (279 total) presented to the emergency department after a suicide attempt during the pandemic period.

**Fig. 2:**
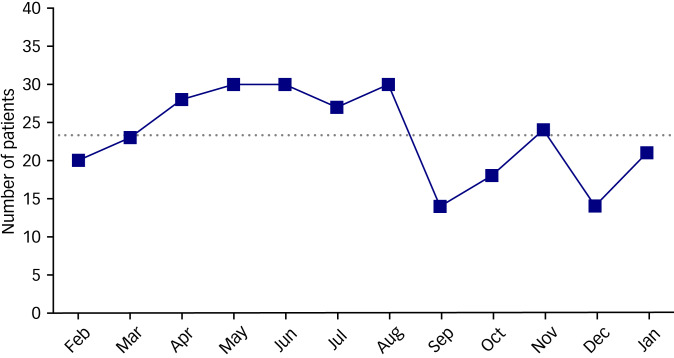
Trends in the number of patients attempting suicide during the pandemic period (February 2020 to January 2021). There was a trend of increasing suicide attempts in the early stage of the pandemic period, but decreasing suicide attempts in the later stage of the pandemic period. The dotted line represents the average number of patients (23.3) per month during the pandemic period.

A total of 581 patients visited our emergency department after a suicide attempt during the study period. Of these, 62 patients with incomplete or missing data were excluded. In total, 519 patients were included in this study. An average of 25.3 patients per month (303 total) in the pre-pandemic period and 23.3 patients per month (279 total) in the pandemic period presented with suicide attempts (Fig. 1). The number of people who attempted suicide increased in the early stages of the COVID-19 pandemic, but decreased in the second half of the pandemic (Fig. 2). The proportion of patients attempting suicide among all patients during the pre-pandemic and pandemic periods was 0.5% and 0.8%, respectively (*P* < 0.001). The median patient age was 30.5 years (IQR, 22–46 years) and 28 years (IQR, 22–48 years) for the pre-pandemic and pandemic periods, respectively. The proportion of women was higher during the pandemic period (69.3%) compared with the pre-pandemic period (60.1%) (*P* = 0.035). The proportion of religious people who attempted suicide was lower during the pandemic (*P* = 0.027). A total of 183 patients (72.9%) had a psychiatric history during the pandemic, whereas 170 patients (63.4%) had a psychiatric history during the pre-pandemic period (*P* = 0.006). During the pandemic period, patients were less likely to attempt suicide when inebriated (*P* = 0.043) (Table 1).

**Table 1:** Baseline demographics and clinical characteristics of the patients who attempted suicide

	Pre-pandemic (*n* = 268), *n* (%)	Pandemic (*n* = 251), *n* (%)	*P*-value
Age, years, median (interquartile range)	30.5 (22.0−46.0)	28 (22.0−48.0)	0.977
Gender, female	162 (60.4%)	174 (69.3%)	0.035*
Employed	102 (33.7%)	76 (35.2%)	0.394
Religious	47 (15.5%)	23 (8.2%)	0.027*
Living with others	183 (60.4%)	129 (59.7%)	0.819
Education			0.123
Never schooled	7 (2.3%)	0 (0%)	
Elementary school	19 (6.3%)	17 (7.9%)	
Middle-high school	157 (51.8%)	101 (46.8%)	
Undergraduate	58 (19.1%)	50 (23.1%)	
Unknown	62 (20.5%)	48 (22.2%)	
Physical illness	41 (15.3%)	36 (14.3%)	0.060
Previous psychiatric history	170 (63.4%)	183 (72.9%)	0.006*
Unipolar depression	125 (73.5%)	144 (78.7%)	
Bipolar disorder	10 (5.9%)	12 (6.6%)	
Anxiety disorder	7 (4.1%)	6 (3.3%)	
Psychotic disorder	8 (4.7%)	9 (4.9%)	
Other psychiatric disorder	20 (11.8%)	12 (6.6%)	
Previous history of suicide attempt	133 (49.6%)	130 (51.8%)	0.495
Reason for suicide attempt			0.557
Conflict with others	130 (48.5%)	134 (53.4%)	
Psychiatric problem	44 (16.4%)	45 (17.9%)	
Economic problem	41 (15.3%)	34 (13.5%)	
Physical illness	20 (7.5%)	12 (4.8%)	
Other	33 (12.3%)	26 (10.4%)	
Post-emergency department status			0.473
Discharge	214 (79.9%)	204 (81.3%)	
Admission	41 (15.3%)	40 (15.9%)	
Transfer	13 (4.9%)	7 (2.8%)	
Intensive care unit admission	24 (9.0%)	29 (11.6%)	0.385
Death	6 (2.2%)	12 (4.8%)	0.089
Alcohol ingestion	148 (55.2%)	111 (44.2%)	0.043*

* *P* < 0.05 (statistically significant).

**Fig. 3:**
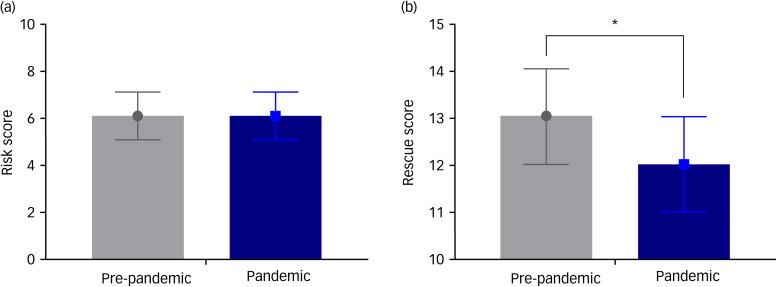
The total risk and rescue scores of patients who attempted suicide during the pre-pandemic (February 2019 to January 2020) and pandemic (February 2020 to January 2021) periods. (a) There was no difference in the total risk score between the pre-pandemic and pandemic periods (both 6; interquartile range, 5–7; *P* < 0.818). (b) The total rescue score of patients in the pandemic period (12; interquartile range, 11–13) was less than that in the pre-pandemic period (13; interquartile range, 12–14) (*P* < 0.001). The solid circle and square represent the median value of the risk score and the rescue score for the pre-pandemic and pandemic periods respectively. The asterisk (*) indicates statistically significant difference.

Regarding the risk factors, patients attempted suicide in a more dangerous way (drowning, asphyxiation, strangulation) during the pandemic period, but suicide attempts by ingestion (such as sleeping pills) and subsequent changes in consciousness were more common during the pre-pandemic period (*P* = 0.009 and *P* = 0.004, respectively). Regarding the rescue factors, the probability of discovery by a rescuer was low and the time lapse between the suicidal behaviour and the start of rescue was also significantly delayed during the pandemic period (*P* = 0.002 and *P* = 0.003, respectively) (Table 2). Figure 3 shows the total risk and rescue scores of the patients who visited our emergency department after a suicide attempt. The risk score for individual patients did not differ between the pre-pandemic and pandemic periods (both 6; IQR, 5–7; *P* < 0.818). The rescue score for individual patients in the pandemic period (12; IQR, 11–13) was less than that in the pre-pandemic period (13; IQR, 12–14) (*P* < 0.001).

**Table 2:** Risk–rescue ratings for suicide assessment of the patients

	Pre-pandemic (*n* = 268), *n* (%)	Pandemic (*n* = 251), *n* (%)	*P*-value
Risk factors			
Agent used			0.009*
Ingestion, cutting, stabbing	236 (88.1%)	201 (80.1%)	
Drowning, asphyxiation, strangulation	19 (7.1%)	39 (15.5%)	
Jumping, shooting	13 (4.9%)	11 (4.4%)	
Impaired consciousness			0.004*
None in evidence	159 (59.3%)	170 (67.7%)	
Confusion, semi-coma	105 (39.2%)	69 (27.5%)	
Coma, deep coma	4 (1.5%)	12 (4.8%)	
Lesions/toxicity			0.748
Mild	177 (66.0%)	165 (65.7%)	
Moderate	76 (28.4%)	68 (27.1%)	
Severe	15 (5.6%)	18 (7.2%)	
Reversibility			0.922
Good	177 (66.0%)	163 (64.9%)	
Fair	67 (25.0%)	63 (25.1%)	
Poor	24 (9.0%)	25 (10.0%)	
Treatment required			0.320
First aid, emergency worker care	221 (82.5%)	209 (83.3%)	
Hospital admission	23 (8.6%)	14 (5.6%)	
Intensive care	24 (9.0%)	28 (11.2%)	
Rescue factors			
Location			0.713
Familiar	224 (83.6%)	207 (82.5%)	
Non-familiar, non-remote	35 (13.1%)	32 (12.7%)	
Remote	9 (3.4%)	12 (4.8%)	
Person initiating rescue			0.559
Key person (acquaintance)	211 (78.7%)	188 (74.9%)	
Professional	43 (46.7%)	49 (19.5%)	
Passer-by	14 (5.2%)	14 (5.6%)	
Probability of discovery			0.002*
High	103 (38.4%)	61 (24.3%)	
Uncertain	158 (59.0%)	184 (73.3%)	
Accidental	7 (2.6%)	6 (2.4%)	
Accessibility of rescue			0.102
Asked for help	164 (61.2%)	132 (52.6%)	
Dropped clues	84 (31.3%)	101 (40.2%)	
Did not ask for help	20 (7.5%)	18 (7.2%)	
Delay until discovery			0.003*
Immediate, 1 h or less	150 (56.0%)	106 (42.2%)	
Less than 4 h	66 (24.6%)	68 (27.1%)	
More than 4 h	52 (19.4%)	77 (30.7%)	

* *P* < 0.05 (statistically significant).

## Discussion

Despite concerns regarding a potential increase in suicide rates during the early stages of the COVID-19 pandemic, a recent study suggested that suicide rates remained constant.^21^ Our results are consistent with this finding. The number of patients who visited the emergency department of our hospital after a suicide attempt during the pandemic period was slightly smaller than those who visited for the same reason during the pre-pandemic period. However, the reduction in suicide attempts should be carefully interpreted. During the pandemic period, we operated the emergency department with a reduced number of beds to reduce the risk of in-hospital infections. Therefore, we were unable to accommodate all patients who visited our emergency department.

The proportion of people who attempted suicide to the total number of patients slightly increased because the total number of patients who visited the emergency department decreased over the COVID-19 pandemic period in comparison with the previous period. Interestingly, the number of people who attempted suicide increased in the early stages of the COVID-19 pandemic, but decreased in the second half of the pandemic. Abrupt environmental changes, such as social isolation and fear of infection, may have affected personal mental health in the early stages of the pandemic.^22,23^ In addition, since public health services were mainly focused on preventing the spread of infectious disease during this period,^24^ social support through measures such as suicide prevention projects was not properly implemented. These factors may have contributed to the increase in suicide attempts during the early stages of the pandemic. In contrast, people gradually became aware of mental health threats as intensive quarantine was implemented.^25^ Various media frequently highlighted the term ‘Corona Blue’, which refers to the mental health risks during the COVID-19 pandemic. The increased awareness of mental health problems during the later stages of the pandemic would have increased the implementation of social support activities for suicide prevention, which may have led to a reduction in suicide attempts.

We also found that the groups more vulnerable to suicide attempts included women and patients with a history of psychiatric disorders. Dubé et al^26^ reported that women were most vulnerable to suicidal ideation during the COVID-19 pandemic. Women have been suggested to be more susceptible to economic difficulties during the COVID-19 pandemic since they are more likely to be employed part-time or temporarily, and would therefore have been more affected by unemployment or income loss under the social lockdown.^27^ Gender inequality in the labour market has been observed in several countries.^28^ Family life was also affected by school closures and telecommuting during the COVID-19 pandemic. Regardless of the overall positive or negative effect of staying at home on family members, these conditions would have increased the burden on women in caregiver roles and increased the scope for conflict in families. One study reported an increase in family violence associated with the COVID-19 pandemic.^29^ Economic and social burdens adversely affect women's mental health. In addition, depression was more prevalent in women even before the COVID-19 pandemic. A previous study reported that the aggregate prevalence of depression was 14.4% for women and 11.5% for men.^30^ The gender difference in the prevalence primarily stemmed from biological differences.^31^ However, their underlying depression may have worsened by factors such as disconnection of interpersonal relationships or increased anxiety during the pandemic period. Therefore, the COVID-19 pandemic would have made the mental health deterioration of women suffering from depression more noticeable.

We showed that people with previous psychiatric illnesses attempted suicide more often during the COVID-19 pandemic. Recent studies suggest that the COVID-19 pandemic has had a serious impact on mental health.^22,23^ In particular, emotional stress and depression may worsen in people with previous psychiatric illnesses, because social distancing makes it difficult for them to receive social support. The vague fear of COVID-19 exposure in hospitals also affected their medical facility usage patterns, and the use of medical facilities was reported to have reduced significantly during the COVID-19 pandemic.^32^ The factors may have also adversely affected the mental health of the patients. External stress and a lack of social and medical support may have led to the worsening of their underlying psychiatric illness.

We also assessed patients with the Risk–Rescue Rating Scale, and the patients in the two periods showed differences in the probability of discovering suicide attempts and the delay until the attempt was identified. Reduced communication with family or friends as a result of social distancing can hinder the identification of suicide attempts and cause delays in rescue after the suicide attempt. If the probability of discovery is low and rescue is delayed, the patient may show a worse prognosis even with the same degree of injury. Our study showed that the mortality and intensive care unit admission rates were high in people who had attempted suicide during the pandemic, although this difference was not statistically significant. Another study also demonstrated that the severity of suicide attempts was high during the COVID-19 period.^33^

However, the association between the COVID-19 pandemic and suicide remains inconclusive. One study reported that the suicide rate increased during the pandemic,^26^ whereas another reported that it did not.^21^ Negative factors in the external environment that threaten mental health and positive factors that increase concern and awareness of mental health threats coexist. Additional studies are required to verify this relationship. This study aimed to determine the characteristics of people who had attempted suicide during the COVID-19 pandemic. The findings highlighted the importance of recognising suicidal ideation and behaviour, because suicide attempts are an important risk factor for suicide.^34^ Although no increase in suicide attempts was noted during the pandemic period, the findings confirmed that women and people with previous psychiatric diseases are more vulnerable to suicide attempts. In addition, we confirmed that suicide attempts that occurred during the pandemic period were more difficult to discover and time to rescue was longer.

Strategies to prevent suicide in vulnerable people with previous psychiatric illnesses and women are essential to help such individuals cope with devastating conditions such as the COVID-19 pandemic. These strategies would include medical and social support that facilitates psychiatric healthcare services and reduces social stressors. Cognitive–behavioural therapy (CBT) is effective in alleviating symptoms of psychiatric disorders related to an increased risk of suicide, such as depression and anxiety.^35^ It is worth considering the introduction of online psychoeducation to avoid face-to-face contact. Internet-based CBT would be a good strategy to reduce psychological problems and reduce the risk of spreading infections during the COVID-19 pandemic.^36^ Some studies have demonstrated the feasibility of an internet-based CBT application to improve the mental health of groups vulnerable to psychological distress during the COVID-19 pandemic.^37,38^ In addition, maintenance of social linkages with these individuals can reduce the threat of suicide, even if social distancing or isolation is inevitably necessary. Intense public mental health activities to keep in touch with vulnerable people during social crises can increase the possibility of discovery of suicide attempts, and facilitate timely rescue of these individuals.

This study had several limitations. First, it had a retrospective design. We could not control for potential confounding factors other than the COVID-19 pandemic, which influenced our results. Second, this study was conducted at a single centre with a small sample size. Therefore, the results of the present study cannot be generalised. Third, we collected data from participants during the early period of the COVID-19 pandemic. The COVID-19 pandemic began in 2020 and has been ongoing for more than 2 years. The number of COVID-19 infections was not large in the early period in Korea. However, fear of new diseases has been severe since the beginning. Quarantine measures have also been implemented. Thus, mental health threats may have persisted since the beginning of the COVID-19 pandemic. Nevertheless, the characteristics of people who had attempted suicide over the entire pandemic period may differ from those in the early period. Further studies are needed to investigate the long-term impact of the COVID-19 pandemic on suicide. Finally, this study only investigated the patients who visited the emergency department after a suicide attempt. Therefore, we could not assess the potential suicidality during the pandemic period. Screening of an individual's potential for future suicidal behaviour in communities, using an instrument such as the Suicidal Affect-Behaviour-Cognition Scale,^39^ would help identify people at risk of suicide during the pandemic period.

In conclusion, continuous monitoring and appropriate suicide prevention efforts for vulnerable groups, especially women and people with previous psychiatric illnesses, are essential to prevent suicides during the COVID-19 pandemic. In addition, social suicide prevention policies and support should be an important priority for coping with similar future crises.

## Data Availability

The data used to support the findings of this study are available from the corresponding author, J.W.K., upon reasonable request.
